# Microbiota as the unifying factor behind the hallmarks of cancer

**DOI:** 10.1007/s00432-023-05244-6

**Published:** 2023-08-09

**Authors:** Iva Benešová, Ľudmila Křížová, Miloslav Kverka

**Affiliations:** 1 Laboratory of Cellular and Molecular Immunology, Institute of Microbiology v.v.i., Czech Academy of Sciences, Vídeňská 1083, 142 00 Prague 4-Krč, Czech Republic; 2 Department of Oncology, First Faculty of Medicine, Charles University in Prague and General University Hospital in Prague, Prague, Czech Republic

**Keywords:** Gut microbiota, Hallmarks of cancer, Tumor microenvironment, Fecal microbiota transplantation

## Abstract

The human microbiota is a complex ecosystem that colonizes body surfaces and interacts with host organ systems, especially the immune system. Since the composition of this ecosystem depends on a variety of internal and external factors, each individual harbors a unique set of microbes. These differences in microbiota composition make individuals either more or less susceptible to various diseases, including cancer. Specific microbes are associated with cancer etiology and pathogenesis and several mechanisms of how they drive the typical hallmarks of cancer were recently identified. Although most microbes reside in the distal gut, they can influence cancer initiation and progression in distant tissues, as well as modulate the outcomes of established cancer therapies. Here, we describe the mechanisms by which microbes influence carcinogenesis and discuss their current and potential future applications in cancer diagnostics and management.

## Introduction

Human gut microbiota forms a complex ecosystem that consists of more than 1000 species of bacteria, over 140 thousand viruses, mostly bacteriophages, and a less diverse ecosystem of archaea and fungi (Camarillo-Guerrero et al. 2021; Kim et al. 2020; Hoffmann et al. 2013; Qin et al. 2010). Studies using gnotobiotic (i.e., germ-free (GF) or artificially colonized) animals clearly showed that without this complex ecosystem, the immune system and many other physiological functions would never reach their full potential (Kverka and Tlaskalova-Hogenova 2017). Each one of us houses more than 10^14^ gut bacterial cells that code 150 times more genes than the human genome. Interestingly, only about one third of these bacterial taxa are shared among individuals (Qin et al. 2010; Curtis et al. 2012). The resulting specific set of abilities may endow the host with a unique metabolic apparatus, which is not coded in their genome and can be, therefore, change with the environment. The “adult” form of microbiota is established during the first 3 years of life and is largely shaped by the environment (Yatsunenko et al. 2012). Throughout life, a dynamic balance of microbial species and their interactions with the host is maintained, but it may be disrupted by diet, diseases or drugs (Zheng et al. 2020a).

Due to the specific environment in the gut, 95% of all bacteria in the typical colonic microbiota of healthy humans belong to four phyla—59% are Firmicutes, 24% are Bacteroidetes, 9% are Actinobacteria, and 3% are Proteobacteria (Bajer et al. 2017). Although all these microbes account for the majority of gut bacteria, the exact composition is highly variable even among healthy people. But the total metabolic activity of this microbial consortium is very similar between individuals and reflects the specificities of the gut niche (Curtis et al. 2012). The gut of healthy adults also contains 10^13^ fungal cells, belonging mainly to the genera *Saccharomyces, Candida,* and *Cladosporium* (Hoffmann et al. 2013). However, the persistent symbiotic inhabitants are only a minority, and most fungal species found in human intestinal isolates are transients and contaminants. It is unclear whether this lack of fungal colonization is a natural condition or the result of morphology changes in the gut during primate evolution or changes in the diet of modern humans, but several diseases are now associated with increased fungal colonization of the gut (Auchtung et al. 2018).

Dysbiosis (i.e., an alteration in the composition and function of the microbiota) is associated with a variety of inflammatory, autoimmune, metabolic, and neoplastic diseases (Lazar et al. 1830). It typically contains one or more of the following non-mutually exclusive characteristics: an overgrowth of potentially pathogenic commensals (pathobionts), a loss of commensals, or a loss of microbial diversity (Levy et al. 2017). However, practical application of this key concept has several important limitations. Although the amount of data on the human gut microbiota is rapidly increasing, there is no generally accepted consensus on what constitutes a healthy gut microbiota, and even the most advanced methods of microbiota analysis have numerous conceptual, technical, and interpretational issues (Kverka and Tlaskalova-Hogenova 2017). As a result, many researchers now advocate focusing on the mechanistic basis of dysbiosis, rather than cataloging the changes in individual microbial species (Tiffany and Baumler 2019).

Nevertheless, specific microbes (e.g., *Fusobacterium nucleatum*) may downregulate T cell response within the tumor, which could drive carcinogenesis of the gastrointestinal tract (Mima et al. 2015). Gut bacteria may also influence the efficacy and adverse event frequency of anticancer therapy (Frankel et al. 2017; Routy et al. 2018; Vetizou et al. 2015). The gut microbiota enriched in members of the Bacteroidetes phylum renders malignant melanoma patients more resistant to ipilimumab-induced colitis, whereas the microbiota enriched in *Faecalibacterium *spp., and other Firmicutes is associated with more frequent colitis but also longer progression-free survival (Chaput et al. 2017; Dubin et al. 2016). This ability of certain Firmicutes to accelerate anticancer response after their translocation through damaged gut mucosa has already been suggested in preclinical studies, showing the mechanistic link between adverse and therapeutic effects (Viaud et al. 2013). These results collectively show that while only some mechanisms are currently known, gut microbiota is not only involved in etiology or pathogenesis of some cancers, but it may be also employed in cancer diagnostics, therapy, and management.

## Microbiota in cancer etiology

Approximately 15–20% of cancers worldwide are caused by or associated with an infectious agent. These pathogens are mainly viruses and parasitic worms that infect a target tissue or compromise immune surveillance, but some bacteria colonizing the gastrointestinal tract mucosa are also implicated in carcinogenesis (IARC 2012). Stomach colonization with *Helicobacter pylori* increases the risk of gastric ulceration (Marshall and Warren 1984), which leads to gastric cancer through long-term irritation and chronic inflammation of the mucosa. This process usually takes several years before the cells accumulate enough genetic aberrations for cancer initiation, and only 1–3% of infected individuals develop gastric adenocarcinomas (Wroblewski et al. 2010). Interestingly, transferring gastric microbiota free from *H. pylori* from gastric patients to mice can induce premalignant lesions (Kwon et al. 2021). This suggests that other gastric microbes or gastric microbiota dysbiosis may induce inflammation and metaplasia in the gastric mucosa.

Patients with colorectal cancer (CRC) have a different gut microbiota than healthy individuals, and the presence of certain microbes in tumor tissue is associated with more advanced tumors (Mira-Pascual et al. 2015). Fusobacteria, particularly *F. nucleatum*, are more abundant in colorectal cancer tissue than either in healthy colon tissue from healthy individuals or in a healthy part of colon from CRC patients (Castellarin et al. 2012; Kostic et al. 2013). Its presence correlates with high microsatellite instability in colorectal cancer patients, suggesting that it may also disrupt the DNA structure (Okita et al. 2020). While these finding are well established, it is not clear whether *F. nucleatum* drives the CRC etiology or whether its presence is due to the favorable environment in the tumor. *F. nucleatum* drives proliferation and invasive activity of colorectal cancer cells by accelerating tumor-promoting inflammation through activation of the nuclear factor kappa-light-chain-enhancer of activated B cells (NF-κB) via the toll-like receptor (TLR) 4—miRNA-21 signaling pathway (Yang et al. 2017).

*Porphyromonas gingivalis* is associated with progressive periodontal disease (Slots et al. 1986), and severe chronic periodontitis is a risk factor for CRC, lung cancer, and oral squamous cell carcinoma (Tezal et al. 2009; Michaud et al. 2018). This G^−^ anaerobe makes cancer cells to proliferate and invade surrounding tissues (Inaba et al. 2014; Geng et al. 2017). This invasion is exacerbated by activation of matrix metalloproteinases mediated by NF-κB activation during tumor-promoting inflammation (Inaba et al. 2014). All three examples of bacteria associated with cancer have the same ability to cause chronic infections and stimulate local pro-inflammatory processes.

Microbes influence carcinogenesis by directly damaging DNA, interfering with DNA repair mechanisms, or promoting inflammation (Ray and Kidane 2016). Production of toxic compounds is a well-established mechanism of how some microbes promote carcinogenesis, but impaired carcinogen elimination could also lead to cancer by increasing an individual’s exposure to carcinogens (Klimesova et al. 2013). Inflammation plays a dual role in cancer, as it can either promote or block tumor growth, depending on the stage of tumor development (Morgillo et al. 2018). This means that the microbiota can drive cancer-promoting genome instability and tumor-promoting inflammation and immunosuppression. Thus, the microbiota influences all cancer cell characteristics, which are referred to as “hallmarks of cancer” (Hanahan and Weinberg 2011; Hanahan 2022).

## Microbes drive hallmarks of cancer

Since Hanahan & Weinberg published their update on the hallmarks of cancer in 2011 (Hanahan and Weinberg 2011), most of the underlying mechanisms became linked to host–microbe interactions. These advances were enabled mainly by the “big data” generated by the current “-omics” approaches. Hallmarks of cancer thus serve as a suitable intellectual framework connecting these new results with specific biological mechanisms. With additional research, new hallmarks and enabling characteristics are emerging (Hanahan 2022), but it seems that all of them may have a common denominator—the microbiome. Here, we describe the main mechanisms of how microbiota influences the hallmarks of cancer and their enabling characteristics.

### Uncontrolled proliferation and spread

The ability to proliferate without regard for surrounding tissues is a major trait of cancers. This ability is based on several cancer cell characteristics that fall into three interconnected groups: sustained and unregulated proliferation, evasion of growth control, and spread throughout the organism.

Microbiota accelerates gut epithelium renewal rate across animal species, as the proliferation of epithelial cells in the gut of GF organisms is considerably slower than in their colonized counterparts (Broderick et al. 2014; Abrams et al. 1963). Additionally, stimulation of the NOD2 receptor by muramyl dipeptide, which is common to all bacteria, triggers intestinal crypt stem cell survival (Nigro et al. 2014). Microbiota regulates 10% of the host’s transcriptome, including genes related to immune response, metabolism, and cell cycle control and proliferation. And while ileal and colonic crypts are regulated differently, microbiota enriches regulatory factors that control cell proliferation in both compartments (Sommer et al. 2015). This suggests that while microbiota modulates epithelial proliferation and differentiation, each gut compartment has distinct requirements for tissue homeostasis. Growth in healthy tissue is strictly controlled by multiple internal suppression systems and signals from the environment. However, cancer cells are insensitive to these signals, evading growth suppression. These signals are further controlled by the Wnt pathway and by activities of the tumor protein p53, both of which are influenced by microbial products.

The Wnt signaling pathway maintains the self-renewal capacity of epithelial stem cells, and its abnormal activity can lead to cancer (Krausova and Korinek 2014). This cascade is critical for cell cycle control, cell migration, apoptosis, and inflammation. Its target genes are important mitogenic sensors that prevent the solid tumor formation in healthy individuals by integrating extracellular mitogenic signals into the cell cycle (Tchakarska and Sola 2020). However, several pathogens have evolved multiple strategies to manipulate the Wnt signaling pathway to increase their ability to infect the host, thereby upsetting this delicate balance (Rogan et al. 2019). Aberrant activation of the canonical Wnt pathway and accumulation of β-catenin in the nucleus is a typical feature of gastric cancer. The CagA protein from *H. pylori* exploits this mechanism to accelerate the proliferation of epithelial cells and inhibit their apoptosis (Song et al. 2015). Similarly, the Cpn1027 protein from *Chlamydia pneumonia* prevents the destruction of β-catenin and thus maintains Wnt signaling in the infected cells in the absence of extracellular stimuli. This increases the expression of the antiapoptotic protein B cell lymphoma-2 (Bcl-2), which prevents the apoptosis of damaged cells (Flores and Zhong 2015). *Ehrlichia chaffeensis* enhances its intracellular survival by regulating epigenetic modification and expression of Wnt genes through the TISS tandem repeat protein (Rogan et al. 2019). The *Bacteroides fragilis* toxin degrades E-cadherin and induces proliferation via the β-catenin pathway (Wu et al. 2003).

The tumor protein p53 protects from cancer by initiating DNA repair, apoptosis, and cell cycle arrest. Its encoding gene (*TP53*) is one of the most frequently mutated genes in human cancers, but its oncogenic potential in the colon remains relatively low in the absence of a specific gut microbial metabolite—gallic acid (Kadosh et al. 2020). To induce cell apoptosis, the tumor protein p53 blocks the activity of the Bcl-2 family antiapoptotic proteins Bcl-xL and Bcl-2. Mutations or posttranslational modifications of any of these proteins can prevent their interaction and interfere with the proapoptotic activity of p53. Colibactin from *Escherichia coli* modifies the tumor protein p53 by SUMOylation, which subsequently induces growth arrest and cancer growth stalling (senescence). However, when the colibactin levels are too low to stop the growth of all tumor cells, senescent cancer cells secrete growth factors that accelerate the growth of surrounding, unaffected cancer cells (Dalmasso et al. 2014). This suggests that the absence or a high dose of colibactin may prevent cancer, while a low dose may promote it.

Cell death due to infection may hinder the ability of some pathogens to multiply in the epithelium and infect the host, prompting them to interfere with cell death signaling. *Shigella flexneri* induces genotoxic stress in the infected epithelial cells, while blocking apoptotic signaling by tumor protein p53 degradation (Bergounioux et al. 2012). Certain commensal gut microbes, such as *Enterobacteriaceae*, drive autocrine production of interleukin (IL)-17C in intestinal epithelial cells through TLR-MyD88-dependent signaling. This, in turn, restricts apoptosis via Bcl-2 and Bcl-xL and promotes cancer cell survival (Song et al. 2014), but IL-17C does not induce proliferation or epithelial–mesenchymal transition (EMT) as does IL-17A (see below). *Enterobacteriaceae*, commensals abundant in dysbiotic gut microbiota, may thus drive cancer by a combination of DNA damage, increased survival, growth promotion, and metastasis.

Cancer can spread (metastasize) to distant tissues via lymphatic and blood vessels. The underlying mechanisms are often associated with inflammation, require proteolytic enzymes, and involve the Wnt pathway. The spread of epithelial tumors depends on the EMT, which is critical for cancer cell migration and invasion of surrounding tissues. Both cancer cells and tumor stromal cells produce a wide range of matrix metalloproteinases (MMPs), most notably MMP2, MMP7, and MMP9 (Jakubowska et al. 2016). Interestingly, IL-17A, which is produced during inflammation, promotes cancer metastasis by inducing EMT in cancer cells via MMP7 (Zhang et al. 2017). This suggests an interesting link between microbiota and EMT, as microbiota induces the expression of IL-17A (see below). Gut dysbiosis is an independent factor that stimulates inflammation and collagen deposition in the tumor and facilitates early metastatic dissemination of hormone receptor-positive mammary cancer in a mouse model (Buchta Rosean et al. 2019). *F. nucleatum* promotes EMT and metastasis formation in CRC by increasing ability of the tumor cells to migrate (Guo et al. 2020; Chen et al. 2020a). On the other hand, gut Bifidobacteria inhibit this process by downregulating the expression of circulating noncoding RNAs (Zhu et al. 2020). Angiogenesis is another important factor in tumor spread. Colonizing the healthy gut of GF mice with microbiota promotes local microvasculature development in the intestinal villi, which is dependent on Paneth cells (Stappenbeck et al. 2002). But in CRC, resident *E. coli* disrupts the gut vascular barrier, allowing bacteria to migrate into the liver, where they create a premetastatic niche and facilitate cancer spread (Bertocchi et al. 2021). Thus, depending on its composition, the gut microbiota can either accelerate or impede the spread of cancer.

### Genome instability and mutations

Genome instability is a tumor characteristic that underlies several hallmarks of cancer. Microbes can weaken genome stability in several ways, either by directly damaging DNA or by disturbing the DNA repair mechanisms. Some microbes produce genotoxins or reactive oxygen species (ROS) and translocating microorganism-associated molecular patterns (MAMPs) promotes chronic inflammation by releasing ROS and reactive nitrogen species (RNS) from resident phagocytes (Aviello and Knaus 2017). These agents then induce single-strand or double-strand DNA breaks, cross-linking of DNA, and mutations, and lead to overall genomic instability (Ray and Kidane 2016), although low levels of ROS are essential for many physiological functions and high levels of ROS kills healthy and cancer cells alike (Lin et al. 2018). One example of a microbe that can significantly contribute to genomic instability is *B. fragilis.* It produces a toxin that can damage the gut barrier, leading to inflammation and increased production of ROS, thus further exacerbating ROS-mediated DNA damage and increasing cancer risk (Haghi et al. 2019). Moreover, this toxin also activates the signal transducer and activator of transcription 3 (STAT3) in intestinal epithelial cells, which leads to proliferation and apoptosis failure and drives chronic inflammation and tumor formation in an IL-17-dependent manner (Wu et al. 2009). Several G^−^ pathogenic bacteria, such as *Campylobacter jejuni*, *Helicobacter hepaticus, Haemophilus ducreyi,* and certain strains of *E. coli,* produce the cytolethal distending toxin. It can contribute to carcinogenesis by inducing genetic instability in replicating epithelial stem cells, which is then passed on daughter cells. Moreover, it induces senescence in CD4^+^ T cells, reducing the efficiency of the immune system’s anticancer response (He et al. 2019; Mathiasen et al. 2021; Tremblay et al. 2021).

The genomic island of polyketide synthase in *E. coli* encodes colibactin. This genotoxin can induce double-strand DNA breaks, which are particularly common in many colon cancers (Dziubanska-Kusibab et al. 2020). Interestingly, microbiota transferred from patients with ulcerative colitis (UC) rapidly induced DNA double-strand breaks in the colon epithelium of mice. *Bifidobacterium infantis* was able to partially counteract this damage by enhancing genomic stability through increased expression of anaphase-promoting complex (APC) 7 in the colon mucosa (Han et al. 2021). APC 7 is associated with a good prognosis in CRC, including higher survival rates and lower cancer recurrence (Kim et al. 2017a). This suggests that both the loss of protective microbes and the presence of genotoxic ones may significantly contribute to the development of colon cancer in patients with inflammatory bowel disease.

### Deregulated cellular metabolism

Tumors require adjustments in energy metabolism to support cell growth and division. Cancer cells exhibit a metabolic anomaly known as the Warburg effect; they rely on a less efficient form of glucose metabolism even in the presence of oxygen—“aerobic glycolysis” (Warburg 1956). This process bypasses the mitochondria and leads to the production of lactate, creating an acidic environment. This shift in pH contributes to tumor progression by inducing genome instability, promoting local invasion and metastasis, and inhibiting anticancer immunity (Ibrahim-Hashim and Estrella 2019).

Microbial metabolites are an essential component of the mammalian systemic metabolome (Wikoff et al. 2009). The gut microbiota can influence host metabolism through signaling molecules, such as short-chain fatty acids (SCFAs), which are produced during the breakdown of complex carbohydrates and proteins (Rios-Covian et al. 2016). The most common SCFAs produced by gut microbiota—acetate, propionate, and butyrate—have different effects on cancer cell metabolism, depending on their concentration. Acetate is an important energy source and key metabolite for growing tumors (Comerford et al. 2014), whereas butyrate slows their growth. Due to the Warburg effect, cancer cells accumulate unmetabolized butyrate, which inhibits histone deacetylases (Donohoe et al. 2012). Thus, microbial metabolites affect epigenetic regulators, especially in cancer cells. SCFAs also affect nutrient metabolism throughout the organism, as acetate is a precursor for hepatic synthesis of monounsaturated long-chain fatty acids, namely palmitoleic acid (16:1) (Kindt et al. 2018). Palmitoleate prevents apoptosis induced by ER stress by interfering with the proapoptotic proteins Bim and PUMA (Akazawa et al. 2010), resulting in cancer cells not responding to the growth-regulatory signals described above. Despite these important effects of microbial metabolites, their use in cancer treatment is still impractical due to the fact that they are produced by diverse gut microbes, have a wide range of activities, and their relative importance in different cancer stages varies. Interestingly, pro-inflammatory cytokines and bacterial toxins are involved in mitochondrial alterations in inflammatory bowel disease (Jackson and Theiss 2020), linking cancer cell metabolism to another important cancer enabling characteristic: tumor-promoting inflammation.

### Tumor-promoting inflammation

Infiltration of immune cells is a well-known feature of the tumor microenvironment in solid tumors (Lanca and Silva-Santos 2012). While these infiltrating cells reflect the immune system’s attempt to eradicate tumors, many of them may promote carcinogenesis by helping tumor cells acquire their characteristic capabilities—to grow and spread. The inflammatory environment also contributes to genetic instability by producing a number of bioactive molecules and creating a vicious circle that promotes further inflammation. This inflammatory tumor microenvironment favors tumor initiation and progression, especially when paired with the immunosuppressive capabilities of malignant tumors (see below).

“"Leaky gut” exposes the intestinal mucosa to excessive amounts of microbial molecules and their toxic products. These molecules further damage the intestinal barrier, increasing the local concentration of pro-inflammatory cytokines and oxidative factors, which in turn damage colonocyte DNA and accelerate tumor-promoting inflammation. MAMPs induce mainly local production of pro-inflammatory cytokines like IL-1β, tumor necrosis factor (TNF)-α, and IL-6 (Schwabe and Jobin 2013). These cytokines drive mesenchymal cells to produce MMP3, further accelerating intestinal damage and failure of the intestinal barrier (Pender et al. 1997). Continued exposure to these pro-inflammatory cytokines and MAMPs activates the NF-κB pathway, leading to prolonged survival of the infiltrating immune cells and prompting them to release additional pro-inflammatory cytokines and oxidative factors (Lombardo et al. 2007; Watson et al. 1998). Similar survival and proliferation signaling are activated in epithelial cells, leading to their neoplastic transformation and local immunosuppression mediated by the programmed death ligand-1 (PD-L1) (Marzec et al. 2008). Similarly to genome instability, these processes are driven by STAT3 activation (phosphorylation), which orchestrates tumor transformation in the cancer cell.

Healthy and cancer cells differ in the intracellular domains of the receptors for pro-inflammatory cytokines, which may elicit different responses (Sheng et al. 2018; Holdbrooks et al. 2018). The typical proapoptotic function of pro-inflammatory cytokines (IL-1β, TNF-α, and IL-6) can actually increase cancer cell survival via mitogen-activated protein kinase/extracellular signal-regulated kinases (MAPK/ERK) or phosphoinositide 3-kinase/ protein kinase B (PI3K/Akt)-dependent pathways (Marques-Fernandez et al. 2013; Wei et al. 2001). TNF-α drives cells into apoptosis at high doses and promotes their survival at low doses, but accumulation of mutations can shift this balance and make cancer cells less susceptible to apoptosis (Li et al. 2009, 2016). During chronic inflammation, the repeated cycle of damage and healing of the intestinal epithelium with low TNF-α levels affects both cancer and immune cells and may keep the inflammation going, while preventing the death of transformed cells. These mechanisms may be responsible for the significantly increased risk of colorectal cancer in UC patients (Hayes 1949). Failure of epithelial barrier function and subsequent low-grade inflammation exacerbated by pro-inflammatory and genotoxic microbial products may also lead to hepatocellular or pancreatic cancers (Cani and Jordan 2018).

Several pro-inflammatory factors also promote angiogenesis, so a local inflammatory environment not only triggers cancer but also promotes its growth and spread. For example, IL-17 promotes vascular endothelial cell migration, cord formation, and regulates the production of several pro-angiogenic factors (Numasaki et al. 2003), while also stimulating the secretion of IL-1β and IL-6 (Li et al. 2016), which are also pro-angiogenic (Hirano 2021; Rebe and Ghiringhelli 2020). Oral probiotics can reduce tumor growth by preventing T helper (Th)17 cells from migrating to the tumor and by limiting the angiogenic effect of IL-17 (Li et al. 2016). However, the role of IL-17 in cancer is highly complex, and the function of IL-17 producing cells in tumors remains poorly understood. Depending on the cancer model, they have been identified as both pro- and anti-tumor cells and are associated with either good or poor prognosis in human cancers, depending on tumor type and stage (Kuen et al. 2020).

The gut microbiota can directly stimulate angiogenesis via its metabolites. For example, nitric oxide, an inducer of angiogenesis, is produced by anaerobic bacteria in the gut in the presence of nitrite or nitrate (Sobko et al. 2005). Moreover, bacterial ligands promote angiogenesis in a TLR- and NOD-like receptor-dependent manner by activating mucosal endothelial and mesenchymal cells (Schirbel et al. 2013). Interestingly, the microbiota of mice with aberrant inflammasome signaling promotes cancer through local secretion of IL-6, which in turn promotes proliferation of epithelial cells and creates a precancerous state (Hu et al. 2013).

This clearly shows that the microbiota can promote carcinogenesis and cancer spread through inflammation. This inflammation is driven mainly by inflammatory cytokines and oxidative factors produced in the tumor microenvironment. To promote tumor growth, rather than prevent it, this inflammation also suppresses the immune response against cancer.

### Avoiding immune destruction

Tumor cells are destroyed mainly by antigen-specific cytotoxic T cells or by pro-inflammatory macrophages stimulated by Th1 cells. Tumors are also targeted by natural killer (NK) cells and even by some antibodies that enable tumor antigen recognition and cell lysis. While immune cells infiltrate solid tumors, oftentimes they are unable to mount an effective immune response. These impaired cells are attracted to the tumor or develop there in response to specific environmental conditions, locally produced cytokines or direct interactions with other cells, including cancer cells.

Cells with immunosuppressive potential, particularly regulatory T cells (Tregs), myeloid-derived suppressor cells (MDSCs), and tumor-infiltrating macrophages (TAMs), dominate the tumor microenvironment of patients with poor prognosis. Signaling through their bacterial or fungal sensors enhances their immunosuppressive activity, which further promotes carcinogenesis (Bayik et al. 2017; Maisonneuve et al. 2021; Wang et al. 2018; Alpdundar Bulut et al. 2020). MDSCs are a heterogeneous population of immature myeloid cells that resemble granulocytes or monocytes and suppress T-cell functions in many inflammatory and neoplastic diseases (Gabrilovich and Nagaraj 2009). These cells are attracted to the tumor environment and converted into immunosuppressive cells by cytokines and tumor cell-derived exosomes (Park et al. 2015; Lin et al. 2016). MDSCs activated in this manner then recruit macrophages to the tumor environment (Lin et al. 2016). Macrophages are highly plastic cells and can adopt any activation state on the continuum between classically activated pro-inflammatory M1 and alternatively activated M2 subtypes (Sica and Mantovani 2012). TAMs are typical M2 macrophages that promote cancer growth and spread by remodeling the tumor stroma, promoting cancer cell survival, inducing neo-angiogenesis, and by suppressing the adaptive immune response (Sica et al. 2006). TAMs produce specific chemokines that actively recruit Tregs to tumors (Liu et al. 2011; Curiel et al. 2004) and the anti-inflammatory cytokine Transforming growth factor (TGF)-β, which stimulates the development of Tregs in tumor tissue (Chen et al. 2003). Tregs not only dampen the anticancer immune response but also decrease IFN-γ by blocking cytotoxic T cells (CTL) and Th1 development (Clark et al. 2020; Liu et al. 2019a), thereby shifting macrophage development toward M2 and closing the vicious circle of immunosuppression. Tregs then promote local immunosuppression by inhibiting local antigen presentation and clonal expansion. They do this by depleting IL-2 using the high-affinity IL-2 receptor, secreting inhibitory molecules such as IL-10, IL-35, and TGF-β or directly killing effector T cells or antigen-presenting cells (Ohue and Nishikawa 2019).

The gut microbiota also regulates anticancer mechanisms, some of which may be disrupted by aberrant host–microbe interactions during early development. Delayed colonization of mice with a complex microbiota increases their susceptibility to colon carcinogenesis later in life through excessive CXCR2-dependent MDSCs accumulation in the tumor (Harusato et al. 2019). However, the microbiota can act as a double-edged sword. Microbial genotoxins can reduce the anticancer response by driving effector CD4^+^ T cells into senescence (Mathiasen et al. 2021), while certain G^+^ microbes translocating from the gut can enhance the anticancer immune response by stimulating the development of IFN-γ^+^ Th17 cells (Viaud et al. 2013). Microbial products, such as butyrate, induce Treg cell differentiation (Arpaia et al. 2013; Furusawa et al. 2013), and while Tregs promote cancer by suppressing the protective immune response, they can prevent the development of colorectal cancer in UC patients by attenuating the chronic inflammation. Some tumors take advantage of the immunosuppressive effects of bacteria by either directly interacting with inhibitory receptors or inducing their expression. For example, *F. nucleatum* inhibits the cytotoxic activities of T cells and NK cells by triggering their inhibitory receptors (Gur et al. 2015, 2019). *H. pylori* attenuates the immune response by inducing PD-L1 expression on epithelial cells and *Mycobacterium bovis* BCG impedes antigen presentation in tumor-infiltrating antigen-presenting cells (Holokai et al. 2019; Copland et al. 2019).

**Fig. 1:**
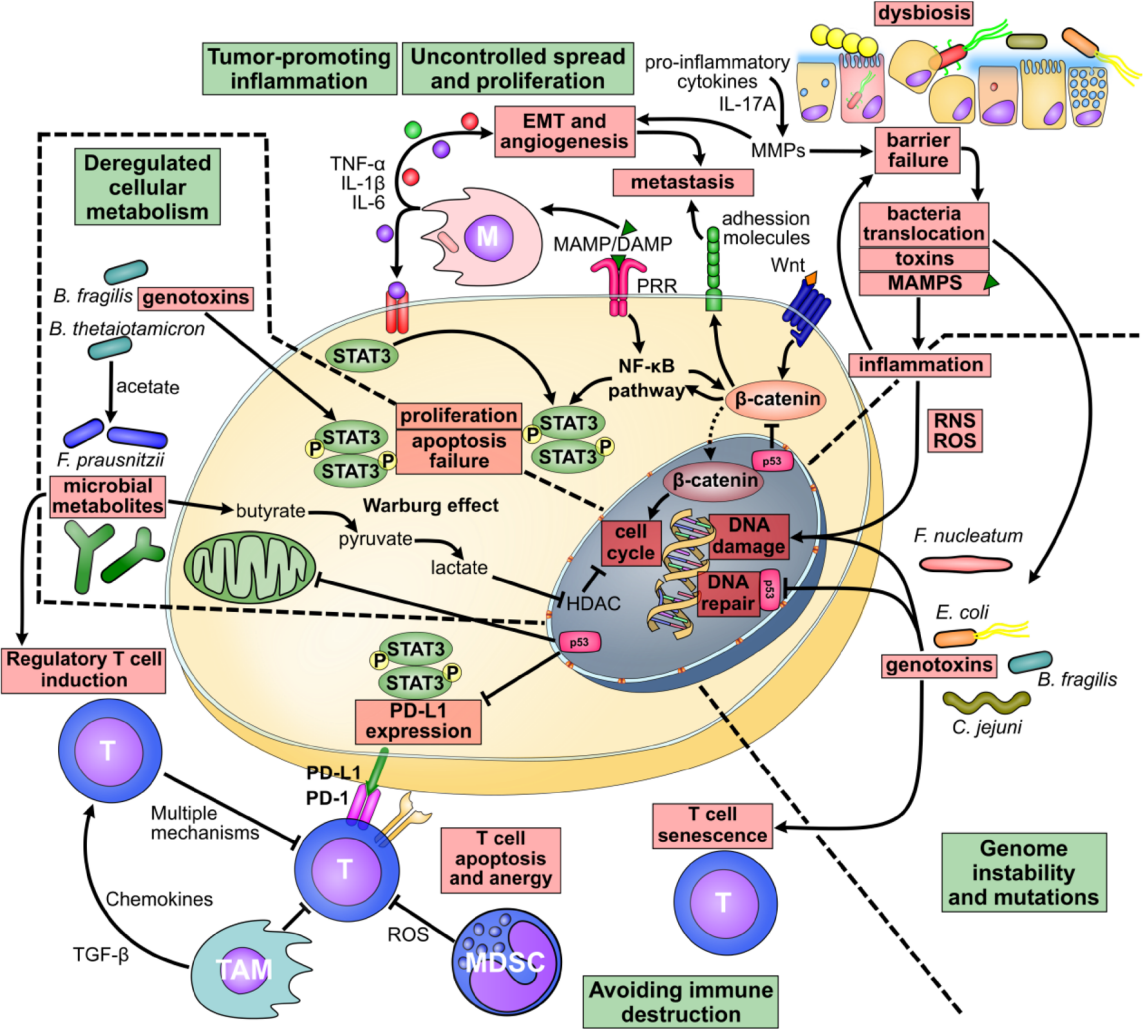
The microbiota, its components, and products link multiple hallmarks of cancer. Leaky gut leads to the translocation of microbes, their components, metabolites, and toxins that damage DNA and its repair mechanisms. They contribute directly (through genotoxins or ROS) or indirectly (through chronic inflammation) to genome instability, deregulated metabolism and uncontrolled proliferation and spread of cancer cells. This affects other cells in the tumor microenvironment and helps cancer cells escape destruction by the immune system and spread to distant sites. Several underlying mechanisms converge in the STAT3 signaling pathway

Taken together, these results suggest that multiple hallmarks of cancer can be triggered by interactions between the host and commensal microbes and that a given microbe or microbial compound can trigger more than one mechanism. Most of these mechanisms are interconnected, resulting in multiple feedback loops (Fig. 1).

## Distant effects of gut microbiota

Disruption of the gut microbiota is a common feature of many cancers and a factor affecting the efficacy of their treatment (Routy et al. 2018). It is easy to conceive how direct microbial involvement in toxin production, barrier disruption, or inflammation might trigger carcinogenesis in the gastrointestinal tract. How the gut microbiota may influence carcinogenesis at distant sites is less clear. First, the microbiota modulates immune maturation, and past interactions with certain microbes (e.g., pathogens) alter future immune reactivity. Second, microbial components and metabolites absorbed from the gut can reach distant tissues and organs. This often depends on the function of the intestinal barrier, as a loose or damaged barrier can greatly enhance this effect. And third, the recirculation of immune cells from the gut mucosa throughout the body ensures the propagation of this effect.

A well-developed microbial ecosystem in the gut protects against colonization by potentially pathogenic bacteria through mechanisms collectively known as colonization resistance. These mechanisms include the stimulation of immune system development, competition with pathogens for resources, and direct combat using antibacterial peptides and bacteriocins (Sorbara and Pamer 2019). Microbes enhance the epithelial barrier functions by improving tight junction formation or increasing the production of mucus and antimicrobial peptides (Ewaschuk et al. 2008; Takiishi et al. 2017). While some microbes have larger impact than others, the very presence of microbes markedly alters the architecture of lymphoid tissue (Round and Mazmanian 2009). In GF animals, intraepithelial lymphocytes are reduced, secretory IgA levels are decreased, and levels of intestinal type 3 innate lymphoid cells and Th17 cells are very low (Zheng et al. 2020a). Signals derived from gut microbes shape the reactivity and antigen repertoire of immune cells and make the immune response more efficient. They alter the development of T cell subsets and even the formation of commensal-specific memory T cells, which can be protective during infections and contribute to the active mechanism of tolerance (Belkaid et al. 2013; Hegazy et al. 2017).

Some T cells in pancreatic tumors from long-term survivors respond to both tumor neoantigens and homologous noncancer microbial antigens (Balachandran et al. 2017). The immune system is able to tolerate neoantigen-mimicking bacteria in the gut while promoting an anticancer inflammatory response to the same antigens in the tumor (Boesch et al. 2021). Interestingly, although microbial colonization rapidly corrects the weak protective immune responses of GF animals (Hapfelmeier et al. 2010), the absence of microbial stimuli early in life may later lead to regulatory mechanisms failure (Hansen et al. 2012). However, prior bacterial infections also alter immune reactivity. Primary pneumonia triggers tolerogenic training of mouse alveolar macrophages and incapacitates them for several weeks (Roquilly et al. 2020). Similarly, acute gastrointestinal infection by *Yersinia pseudotuberculosis* induces chronic mesenteric lymphadenopathy, derails the migration of dendritic cells (DCs) from the gut and impairs the protective and tolerogenic functions of mucosal immunity for an extended period (Fonseca et al. 2015). This suggests that interactions with the gut microbiota can reprogram immune reactivity to many threats, including tumors.

The gut microbiota can influence the immune response during carcinogenesis through bacterial metabolites, bacterial extracellular vesicles (BEVs), and other structural components. After crossing the intestinal barrier, all of these factors can reach distant tissues and organs via lymphatic and systemic circulation (Chronopoulos and Kalluri 2020). BEVs are heterogeneous in size and density and contain a diverse mixture of periplasmic peptides, toxins, nucleic acids, peptidoglycans, and other MAMPs. Depending on their cargo, BEVs can drive the immune system toward harmful inflammation, effective anticancer immunity, tolerance, or immunosuppression (Fabrega et al. 2016; Kim et al. 2017b; Lee et al. 2007; Winter et al. 2014). Structural components of gut microbes can translocate out of the gut due to leaky intestinal barrier and alter the immune response in the tumor microenvironment. Large amounts of bacteria or LPS translocated from the gut increase CXCL1 expression in hepatocytes, leading to accumulation of CXCR2^+^ MDSCs and promotion of liver cancer (Zhang et al. 2021). Similarly, TLR5-dependent interaction with the commensal gut microbiota accelerates carcinogenesis at anatomically distant sites through tumor-promoting systemic inflammation and by attracting MDSCs and immunosuppressive γδ T cells into the tumor microenvironment (Rutkowski et al. 2015). Therefore, structural components of gut microbes markedly influence the tumor microenvironment, often in a cytokine-dependent manner.

Microbial metabolites affect multiple mechanisms, some of which may have opposing biological functions. SCFAs produced by gut bacteria from dietary fiber or protein either promote (acetate) or prevent (butyrate/propionate) inflammation. Butyrate both inhibits the immune response by inducing Tregs (Furusawa et al. 2013) and enhances it by increasing IFN-γ production in CD8^+^ T cells in an ID2-dependent manner (He et al. 2021). Acetate can affect the immune response in different ways, depending on the stage of inflammation. Initially, it increases the capacity of memory CD8^+^ T cells for glycolysis and inflammation, but over time, its accumulation inhibits T-cell receptor signaling and thus reduces their anticancer response (Balmer et al. 2020). But SCFAs are not the only microbial products that affect cancer. Gut commensal microbes such as *Bifidobacterium pseudolongum*, *Lactobacillus johnsonii*, and *Olsenella* sp. produce inosine, which is absorbed from the gut, spreads throughout the body, and increases tumor sensitivity to immune checkpoint inhibitors (ICIs). This enhanced anticancer immunity is driven by intratumoral CD4^+^ and CD8^+^ T cells that produce IFN-γ (Mager et al. 2020). Similarly, the peptidoglycan hydrolase SagA produced by *Enterococcus faecalis* generates specific immunoactive muropeptides that activate the peptidoglycan sensor NOD2 and enhance response to immunotherapy (Griffin et al. 2021). These muropeptides may also be absorbed from the gut and influence the systemic immune response (Huang et al. 2019). The gut microbiota influences the metabolism of bile acids in the gut, which has a profound impact on the immune response against cancer. Primary bile acids increase CXCL16 expression in portal blood veins, while secondary bile acids have the opposite effect. This is important in mouse models of primary and metastatic liver tumors because CXCL16 accelerates the accumulation of NKT cells in the liver. Since G^+^ bacteria convert primary bile acids to secondary bile acids, their removal results in a higher accumulation of NKT cells in the liver and reduced tumor growth (Ma et al. 2018).

Gut microbes can populate tumors because the hypoxic nature of the tumor microenvironment is suitable for many gut inhabitants. They can enter the tumor directly from the bloodstream because of leaky blood vessels, or they can be brought there by leukocytes (Lu et al. 2021; Berg and Garlington 1979; Rescigno et al. 2001). A typical intestinal pathogen can translocate from the intestine through M cells (transcellular pathway, such as *S. flexneri*) or breach the integrity of the intestinal barrier (paracellular pathway, such as *Salmonella enterica* serovar Typhimurium) (Rey et al. 2020; Sun et al. 2020). These translocation abilities are closely related to their virulence factors, but many commensal bacteria leave the intestine without causing any obvious adverse effects (Fung et al. 2016; Macpherson and Uhr 2004; Reddy et al. 2007). Microbes can then reach distant tissues and even be transmitted from mother to infant via breast milk (Perez et al. 2007). While milk contains predominately aerotolerant Proteobacteria and Firmicutes, 49% of all microbes in breast milk originate in the intestine (Togo et al. 2019). Commensal bacteria colonize not only the intestinal surface but also lymphoid tissues, where they facilitate tissue-specific immune responses and maintenance of the intestinal barrier (Fung et al. 2016). In addition, DCs residing in the intestine can directly sample the intestinal lumen for bacteria without breaching the integrity of the intestinal barrier and transport the bacteria to the mesenteric lymph nodes (Rescigno et al. 2001). Intestinal DCs can retain small quantities of live commensals for several days (Macpherson and Uhr 2004), offering a possible explanation for the presence of live intestinal commensals in distant tissues. Indeed, microbes within tumors are mainly found inside immune and cancer cells (Nejman et al. 2020). While immune cells may acquire the microbes in the tumors or even transport them from the gut (Morton et al. 2014), it is unknown how these bacteria enter cancer cells.

**Fig. 2:**
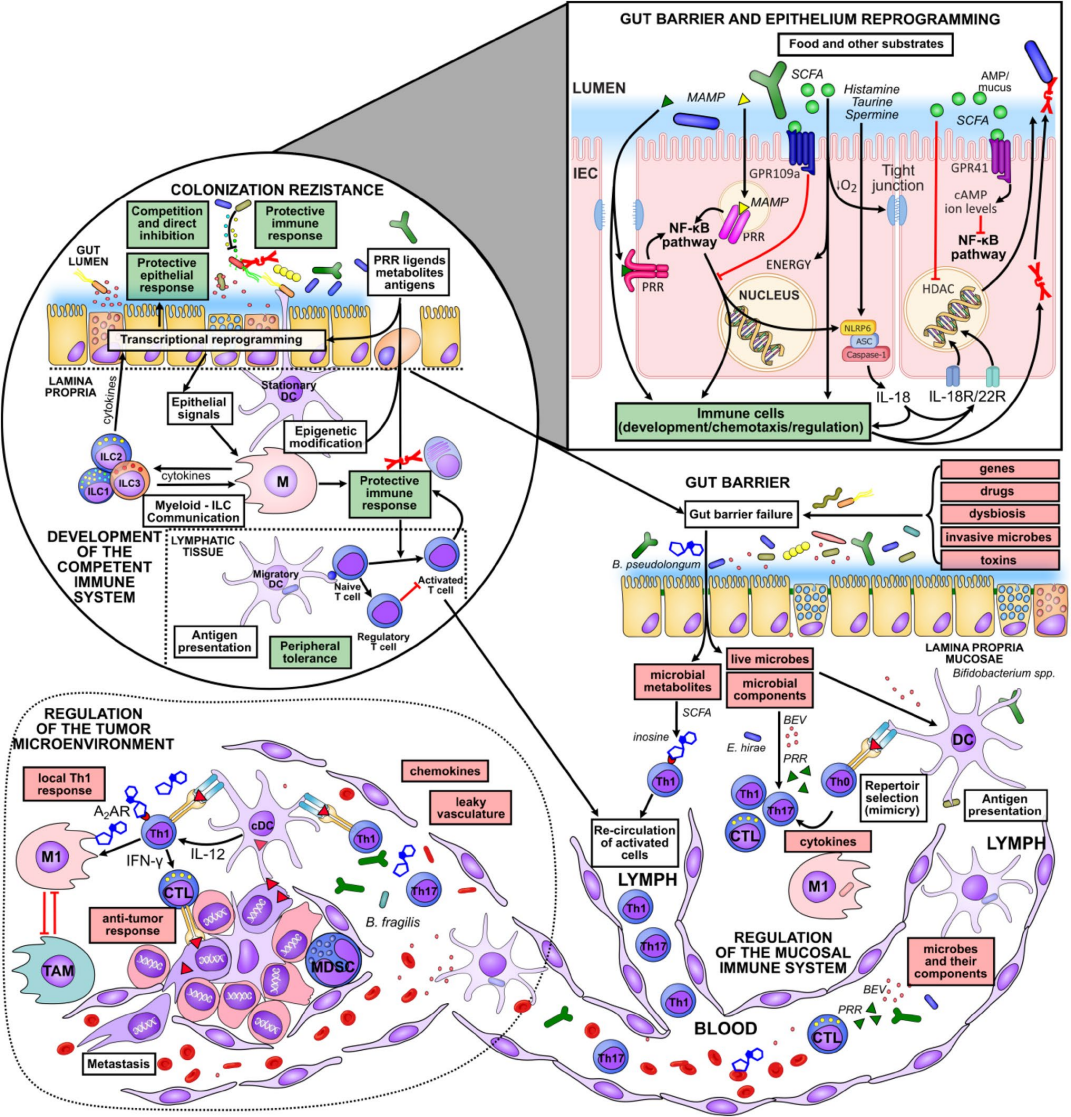
The gut microbiota influences carcinogenesis at distant sites in several ways. First, the gut microbiota modulates the development of the immune system and the gut barrier function. A well-developed gut microbiota prevents colonization by carcinogenic bacteria through a mechanism known as colonization resistance. In addition, foods and other substrates influence the composition of the gut microbiota, reprogramming the functions of the intestinal epithelium. This ultimately alters the resilience of the intestinal barrier and the development, chemotaxis, and regulation of immune cells. Live microbes, their components, and metabolites can enter the tumor through the bloodstream or by transport in the infiltrating immune cells. This effect can be greatly enhanced if the intestinal barrier fails and more microbial components enter the body. Transfected microbes can also alter the reactivity and repertoire of immune cells, which can then migrate to distant tumors and extend the immune response to the entire body

The gut microbiota can influence distant tumors in a variety of ways. This may explain why the gut microbiome is associated with cancers in distant organs, such as the breast, liver, pancreas, or lung (Ma et al. 2018; Goedert et al. 2015; Zheng et al. 2020b; Matsukawa et al. 2021). And while immune system development, leukocyte trafficking, and direct colonization of tumors by carcinogenic bacteria have all been described as important mechanisms by which microbes influence cancer, multiple mechanisms may be at work simultaneously (Fig. 2).

## Microbiota as biomarker

Biomarkers are objective indicators of a medical state that can be accurately and reproducibly measured and thus can be used for diagnostics and outcome prediction. The best biomarkers are directly related to disease etiology or pathogenesis or are produced as a direct consequence of the pathological process. As certain microbes are involved in various processes of carcinogenesis they can serve as suitable biomarkers for predicting a cancer diagnosis, its outcome and therapy efficacy. The source of microbial biomarkers may be the tumor, its proximal tissue, or even an anatomically distant organ, as described above. While several studies have already shown promising results using this approach, some problems have also emerged. It is not clear if these results are universal across different human populations and how they might be affected by confounding factors (e.g., initial colonization, diet, environment, medication, disease stage) or sampling and processing methods. For example, most published studies examined urban populations from the so-called WEIRD (Western, Educated, Industrialized, Rich, and Democratic) countries (Gupta et al. 2017), but rural and urban lifestyles result in distinct gut and skin microbiomes even within one population (Ying et al. 2015; Obregon-Tito et al. 2015). Even though identifying a universally applicable set of microbial biomarkers may be an unattainable goal, we might be able to link certain predictive biomarkers to specific and easily defined population characteristics.

Because of the close relationship between gut microbiota and CRC, biomarkers from the gut microbiota are often studied in CRC. T*he presence of Faecalibacterium prausnitzii* is associated with a good prognosis (Wei et al. 2016), whereas high abundance of *F. nucleatum* or *B. fragilis* in colon tissue is associated with more advanced tumors and poorer prognosis in CRC patients (Mima et al. 2016; Flanagan et al. 2014). *F. nucleatum* can accelerate tumor progression, making it a suitable biomarker and even a potential therapeutic target. While colon tissue is not well suited for large-scale colorectal cancer screening, both *F. nucleatum* and *B. fragilis* are also enriched in the stool of CRC patients across different populations, suggesting that some fecal microbes may serve as universal CRC biomarkers (Dai et al. 2018).

Cancers of the pancreas and liver are strongly influenced by the digestive tract microbiota, and both the liver and pancreas in turn shape the gut microbiota through their external secretions (David et al. 2014). Specific signatures of the gut microbiota are associated with different types of liver cancer and distinguish them from microbiota of healthy individuals and patients with cirrhosis. They also predict poor prognosis in liver cancer, suggesting that the gut microbiota profile is useful as a biomarker for cirrhosis-related carcinoma (Jia et al. 2020; Ponziani et al. 2019). Patients with pancreatic cancer also show gut dysbiosis, and certain microbes in their feces, saliva, and cancerous tissue are strongly associated with a poorer prognosis (Matsukawa et al. 2021). A recent meta-analysis found that certain periodontal bacteria (e.g., *P. gingivalis* or *Prevotella intermedia*) are associated with increased cancer incidence and even predict poor prognosis in cancer patients (Xiao et al. 2020a). This association is of particular interest in head and neck cancers because the presence of *P. gingivalis* in the oral cavity predisposes to periodontitis, which is a risk factor for several cancers that are in close proximity to the oral microbial community (Galvao-Moreira and Cruz 2016).

The lower female reproductive tract (vagina and cervix) is heavily colonized by microbes (Laniewski et al. 2020). Patients with a vaginal microbiota dominated by *Lactobacillus *spp. have a higher chance of cervical intraepithelial neoplasia regression compared to those with *Lactobacillus*-depleted communities enriched in anaerobes (Mitra et al. 2020). This form of dysplasia is often caused by human papillomavirus (HPV) infections, and while HPV infection is associated with increased diversity of the vaginal microbiota, both HPV and increased diversity are independent risk factors for progression of cervical dysplasia (Chen et al. 2020b; Mitra et al. 2015). The strain of *Lactobacillus* matters, because *Lactobacillus crispatus* dominance is associated with a low prevalence of HPV, dysplasia, and cancer, whereas *Lactobacillus iners* dominance is not (Norenhag et al. 2020). This indicates that *L. crispatus* is a suitable biomarker for good prognosis in HPV-positive patients with cervical intraepithelial neoplasia and may even suggest a potential probiotic supplementation strategy for patients without *L. crispatus*.

The vaginal microbiota represents an important regional source of microbiota and might also provide biomarkers for other gynecologic malignancies. Similarly to cervical cancer, a lactobacillus-poor vaginal microbiota is associated with ovarian cancer, especially in younger women (Nene et al. 2019; Morikawa et al. 2022). In addition, infections with *Chlamydia trachomatis* or *Mycoplasma hominis* increase the risk of ovarian cancer, and cancerous ovarian tissue contains more *Brucella*, *Chamydia*, and *Mycoplasma* than healthy tissue (Xu et al. 2020). It is not clear whether this is a direct consequence of ascending colonization through the fallopian tube or an indirect effect, e.g., immune-mediated. The peritoneum contains only a limited inoculum, which is significantly altered in patients with ovarian cancer. Adding these microbial alterations to standard tumor biomarkers significantly increases their diagnostic potential for ovarian cancer (Miao et al. 2020).

Another interesting example is breast cancer. Breast microbiome has a unique composition, which is quite different from the overlying skin, and women with benign diseases have markedly different breast microbiome than women with malignant disease (Hieken et al. 2016; Urbaniak et al. 2016). Postmenopausal women with breast cancer have less diverse gut microbiota than healthy women, revealing a new link between breast cancer and the gut microbiota (Goedert et al. 2015). The gut microbiota can influence breast cancer in multiple ways. Dysbiosis contributes to obesity, leading to abnormal sex hormone production, and also contributes to hormone reabsorption from the gut by releasing them from their glucuronide bonds (Flores et al. 2012). Gut microbes also possess genes coding for estrogen-metabolizing enzymes called the estrobolome (Komorowski and Pezo 2020) and similar mechanisms for androgens (Pernigoni et al. 2021). Thus, the gut microbiota may also serve as a relevant biomarker for other types of sex hormone-dependent malignant diseases, such as ovarian, endometrial, and even prostate cancers (Qi et al. 2021).

Lung cancer can be predicted by changes in local or distant microbial communities. The airway microbiota has a specific composition in patients with lung cancer, both airway and gut microbiota dysbiosis is associated with lung cancer (Perrone et al. 2021; Liu et al. 2019b), and a specific signature of the gut microbiome may even predict early-stage lung cancer (Zheng et al. 2020b). In non-small-cell lung cancer (NSCLC), the gut microbiota has repeatedly been shown to be a suitable biomarker for response to ICIs (see below), suggesting that it is the immune system that connects the gut to the lung. Another interesting example of a similar long-range interaction is the correlation of melanoma invasiveness with gut microbiota and mycobiota signature (Vitali et al. 2022). Although the mechanisms have not yet been studied in detail, specific microbial signatures associated with good response to ICI in melanoma patients suggest an interesting mechanistic link.

Because of its involvement in carcinogenesis, the microbiota may soon become a cancer biomarker or even a therapeutic target, but before this happens, several issues need to be addressed in future research. First, multiple factors can influence the microbiome, so it is necessary to standardize sampling and take confounding factors into account. Second, the shifts in the microbiota observed in cancer patients may be caused by the disease itself or by its treatment, which is particularly pertinent for microbial communities in close proximity to tumors. Thus, early diagnostic studies may require a different approach than those aimed at predicting disease progression. The fundamental role of the gut microbiota in all main types of cancer therapies has been recently explored with promising success.

## Microbiota as therapeutic modulator

Interindividual differences in gut microbiota composition translate into variability in anticancer drug efficacy and toxicity. The gut microbiota modulates host response to anticancer drugs through mechanisms related to translocation, immune modulation, metabolism regulation, enzymatic degradation, and by reduced diversity and ecological variation (Alexander et al. 2017). Therefore, targeting the gut microbiota to match patients to a specific therapeutic option or to personalize therapy according to the composition of the microbiota is a viable approach to improve the efficacy and reduce the toxicity of current cancer therapies. Intact gut microbiota may be needed to mobilize the immune system, so antibiotics (ATBs) may indirectly affect the efficacy of anticancer drugs. However, ATBs are typically used to address secondary infections. Therefore, it is important to consider that the actual culprits behind cancer treatment failures might be the infectious agents or immune deficiencies themselves.

### Chemotherapy

Chemotherapy damages the intestinal mucosa and causes dysbiosis by affecting the ecology of the gut microbiota (Montassier et al. 2015; Huang et al. 2012). This dysbiosis can be long-lasting, so it can contribute to long-term side effects of chemotherapy (Deleemans et al. 2019). Depending on its composition, gut microbiota can either promote or prevent carcinogenesis and cancer progression. A diverse gut microbiota can improve the efficacy of chemotherapy by promoting a strong cellular immune response against cancer without leading to tumor-promoting inflammation. Cyclophosphamide, for example, enables translocation of selected G^+^ bacteria to lymph nodes by disrupting the integrity of the intestinal epithelium, thereby inducing IFN-γ^+^IL-17^+^ Th cells that can slow down tumor growth (Viaud et al. 2013). Tumors in mice whose gut microbiota is disrupted by ATBs respond poorly to platinum chemotherapy due to impaired respiratory burst in their tumor-infiltrating myeloid cells (Iida et al. 2013). On the other hand, some microbes make chemotherapy less efficient. *F. nucleatum* increases the chemoresistance of colon cancer cells by promoting autophagy (Yu et al. 2017), and some microbes with inefficient ribonucleotide metabolism impair the efficacy of 5-fluorouracil through its metabolic conversion (Scott et al. 2017).

### Immunotherapy

The gut microbiota modulates the efficacy of cancer immunotherapy, and this effect can be transferred to experimental animals through fecal transplants (Routy et al. 2018; Gopalakrishnan et al. 2018; Matson et al. 2018). It is possible that this is due to specific microbial metabolic pathways, *as* melanoma patients who responded to the anti-programmed death 1 (PD-1) immunotherapy had enriched anabolic pathways compared to those who did not respond. Interestingly, these differences were particularly pronounced in the gut microbiome, whereas the oral microbiome of responders differed only slightly from that of non-responders (Gopalakrishnan et al. 2018). The microbial signatures found to date are inconsistent (Table 1), which may be due to differences in cancer types, therapeutic agents, and treated populations. Immunotherapy depends on a functioning immune system, acting synergistically with mechanisms that enhance the immune responses to cancer (Sivan et al. 2015). A specific microbe within the microbiota may influence the response to anticancer treatment (Vetizou et al. 2015; Mager et al. 2020; Sivan et al. 2015), but complex microbial interactions with the entire bacterial consortium may be required to promote a robust anticancer immune response during immunotherapy (Tanoue et al. 2019). Interestingly, although increased consumption of dietary fiber enhances the response to immunotherapy in an experimental animal model of melanoma, the addition of probiotics (*Bifidobacterium longum* or *Lactobacillus rhamnosus* GG) negates this effect (Spencer et al. 2021). This suggests that specific microbes may disrupt the beneficial effect of nutritional factors on ICI efficacy by modulating the anticancer immune response in the tumor microenvironment.

**Table 1:** Association between bacterial abundance and effectiveness of immunotherapy

Cancer type	ICI	Higher in responders	Higher in nonresponders	References
Melanoma	anti–CTLA-4	*Faecalibacterium *spp., Firmicutes	Not observed	Chaput et al. (2017)
Melanoma	anti–CTLA-4 + antiPD-1	*Bacteroides caccae*, *F. prausnitzii*, *Bacteroides thetaiotamicron*, *Holdemania filiformis*	Not observed	Frankel et al. (2017)
Melanoma	anti–PD-1	*Bacteroides caccae*, *Dorea formicogenerans*	Not observed	Frankel et al. (2017)
Melanoma	anti–PD-1	*Ruminococcaceae*	*Bacteroides thetaiotaomicron*, *E. coli*, *Anaerotruncus colihominis*	Gopalakrishnan et al. (2018)
Melanoma	anti–PD-1 or anti–CTLA-4	*Bifidobacterium longum*, *Collinsella aerofaciens*, *Enterococcus faecium*	*Ruminococcus obeum*, *Roseburia intestinalis*	Matson et al. (2018)
NSCLC	anti-PD-1	*Lactobacillus *spp., *Clostridium *spp.	*Sutterella *spp., *Bilophila* spp.	Katayama et al. (2019)
NSCLC	anti–PD-1	*Alistipes putredinis*, *B. longum*, *Prevotella copri*	*Ruminococcus* spp. (unclassified)	Jin et al. 2019)
NSCLC	anti-PD-1	*Parabacteroides* spp., *Methanobrevibacter* spp.	*Veillonella* spp., Selenomonadales and other Negativicutes	Song et al. (2020)
NSCLC	anti–PD-1 or anti–PD-L1	Firmicutes (especially *Akkermansia muciniphila*) *Ruminococcus* spp.* Alistipes* spp. *Eubacterium* spp.		Routy et al. (2018)
NSCLC	anti–PD-1 or anti–PD-L1	*A. muciniphila* (low abundance), *Eubacterium hallii*, *Bifidobacterium adolescentis*	Either missing or high abundance of* A. muciniphila*	Derosa et al. (2022)
Renal cancer	anti–PD-1 or anti–PD-L1	Firmicutes (especially *A. muciniphila*)		Routy et al. (2018)
Renal cancer	anti-PD-1 or anti–CTLA-4 + antiPD-1	*A. muciniphila*		Salgia et al. (2020)

Different microbial communities distinguish patients who respond to various ICI therapies from those who do not. Melanoma patients who responded to the combination of the cytotoxic T-lymphocyte-associated protein 4 (CTLA-4) blocker ipilimumab and the PD-1 blocker nivolumab had a gut microbiome with increased abundances of *F. prausnitzii, Bacteroides thetaiotamicron*, and *Holdemania filiformis*, whereas patients who responded to the PD-1 blocker pembrolizumab had a gut microbiome enriched in *Dorea formicogenerans*. A higher abundance of *Bacteroides caccae* was common to both groups of responders (Frankel et al. 2017). *Faecalibacterium* and other *Ruminococcaceae* and enrichment of B vitamin metabolism are typical features in responders to immunotherapy, whereas *B. thetaiotaomicron*, *Adlercreutzia equolifaciens*, *Bifidobacterium dentium*, and *Mogibacterium* spp. and an enrichment of aerobic metabolism pathways are typical for gut microbiota of non-responders (Limeta et al. 2020). There is also an association between a specific microbe, high efficacy of ICI and its adverse effects. Melanoma patients with high abundance of *Faecalibacterium* sp. and other Firmicutes in their gut microbiota respond better to the CTLA-4 blocker ipilimumab, but also have a higher incidence of immunotherapy-related colitis (Chaput et al. 2017). This may not be a universal feature, because in some cohorts high levels of fecal *Faecalibacterium* is associated with worse prognosis in patients with malignant melanoma and fecal butyrate limits the anticancer effect of CTLA-4 blockade (Coutzac et al. 2020).

ATBs have a negative effect on treatment outcomes of ICIs in solid tumors (Elkrief et al. 2019). This effect is much weaker if the ATBs were administered a longer time before the start of immunotherapy (Derosa et al. 2018). However, only a handful of studies with small sample sizes investigated this interaction, so their results are difficult to generalize beyond the specific combination of the antimicrobial spectrum and the type of tumor. Broad-spectrum ATBs against anaerobes and intravenous vancomycin have the most detrimental effects on therapy in NSCLC patients, whereas penicillins and early-generation cephalosporins severely impair the treatment of renal cancer (Kulkarni et al. 2020; Derosa et al. 2022). The mechanisms behind the procarcinogenic effects of ATBs are currently not fully understood.

### Radiotherapy

Radiotherapy (RT) is an essential part of the therapeutic armamentarium in oncology. Because ionizing radiation damages all living matter, RT is associated with marked changes in the microbiota in both humans and animal models (Kalkeri et al. 2021; Kim et al. 2015; Nam et al. 2013). This effect could be caused either by direct damage to the microbiota by the radiation (in the exposed regions) or by physiological changes in the patient. These effects may be long-lasting and may thus directly or indirectly modulate RT efficacy and gastrointestinal toxicities. Therefore, manipulation of the microbiota with probiotics, prebiotics, and ATBs can be used as an additional therapeutic measure (Liu et al. 2021; Tonneau et al. 2021).

Dysbiosis caused by broad-spectrum ATBs or multiple administrations of ATBs is associated with unfavorable outcomes of RT in locally advanced head and neck cancer. However, it is difficult to determine which therapeutic modality is affected because RT is usually supplemented with chemotherapy in these cases (Nenclares et al. 2020). In patients with rectal cancer treated with preoperative chemoradiotherapy, the specific signature of the gut microbiota, but not alpha diversity, distinguishes between complete and incomplete responders. While *Duodenibacillus massiliensis* is enriched in the former, the gut microbiota of the latter *is* associated with the *Bacteroidaceae* family, particularly *Bacteroides* spp. and *Rikenellaceae* spp (Jang et al. 2020). Diverse gut microbiota is associated with favorable outcomes in patients with cervical cancer treated with brachytherapy. This effect is probably indirectly driven by the immune system, as high diversity of the gut microbiota correlates with CD69^+^, Ki-67^+^, or PD-1^+^ tumor-infiltrating CD4^+^ T lymphocytes (Sims et al. 2021), which are also associated with longer survival in head and neck squamous cell carcinoma (Badoual et al. 2006). This may be due to the redundancy of immunomodulatory properties of the gut microbiota, as a highly diverse microbiota is more likely to contain biologically active microbes.

The gut microbiota is an important determinant of sensitivity to RT-related colitis, as dysbiosis reduces the gut barrier resistance, allowing microbes to translocate and triggering an inflammatory response in the intestinal mucosa (Liu et al. 2021). Early clinical trials have shown that fecal microbiota transplantation (FMT) can alleviate radiation enteritis in humans (Ding et al. 2020). Treatment with oral vancomycin decreases butyrate production in the gut and enhances the anticancer effect of RT in tumor-bearing mice. This synergy depends on cross-presentation of tumor-associated antigens to cytotoxic T cells by DCs in tumor-draining lymph nodes (Uribe-Herranz et al. 2020), indicating that the underlying mechanism depends on the immunomodulatory effects of SCFAs.

Although SCFAs may be associated with worse therapeutic outcomes because of their trophic and immunomodulatory effects, they reduce the severity of radiation-induced damage to the gastrointestinal tract (Denton et al. 2002; Al-Sabbagh et al. 1996). A significant decrease in fecal butyrate was found in prostate cancer patients who experienced gastrointestinal symptoms during radiotherapy, while patients without these symptoms showed no changes in butyrate levels (Ferreira et al. 2021). This suggests that the adverse events may not be related to absolute SCFA concentrations, but to their dynamic changes. Other microbiota-derived molecules also offer protection from radiation-induced toxicity. These include indole-3-propionic acid, which helps to reduce injury to the hematopoietic system and gastrointestinal tract, and L-histidine, which attenuates cardiopulmonary injury (Chen et al. 2021; Xiao et al. 2020b). Therefore, manipulating the gut microbiota can complement and even enhance cancer treatment.

## Manipulations with microbiota

Diet is the most important modulator of the gut microbiota. Changes in substrate availability drive microbiota adaptation that fundamentally alters the gut microbial ecosystem (Muegge et al. 2011; Wu et al. 2011). Short-term changes in diet or periodic changes in nutrient availability can also trigger reproducible changes in the microbiota (Thaiss et al. 2014; Wang et al. 2017). These changes are caused not only by the presence of specific substrates but also by the organism’s response to these substrates, such as bile production (David et al. 2014). For instance, in the presence of high amounts of animal protein, gut microbes preferentially conjugate bile acids with taurine, which can then be used by sulfidogenic gut bacteria to produce inflammatory and genotoxic metabolites (Ridlon et al. 2016). In addition, processed foods contain other ingredients besides nutrients. Emulsifiers (e.g., carboxymethylcellulose or polysorbate-80) and other food additives promote carcinogenesis through microbiota-dependent low-grade inflammation of the colon in an animal model of colorectal cancer (Viennois et al. 2017). Dietary components can trigger cancer by modifying the microbiota, serving as a substrate for the production of bioactive molecules, and participating in biological signaling.

Dietary factors have long been associated with cancer (Doll and Peto 1981). The consumption of red meat or meat processed by salting, curing, fermenting, smoking, or similar methods is considered carcinogenic in humans (IARC 2018; Veettil et al. 2021). Although human studies frequently yield inconsistent results, raising major criticism and doubts about such associations (Michels 2005), large studies of heterogeneous populations have clearly demonstrated numerous links between cancer, dietary, and lifestyle factors. Obesity is one of the most important factors associated with high caloric intake and cancers, including early-onset colorectal and breast cancers (Ellingjord-Dale et al. 2021; Li et al. 2021). However, it is still unclear whether this epidemiological observation is primarily due to the high energy intake, the low-grade chronic inflammation associated with obesity, or underlying shifts in the gut microbiota.

The gut microbiota can be transferred from a healthy donor, directly impacting the gut microbial community of the recipient. FMT is used to restore gut microbial ecology in patients with *Clostridioides difficile* infection (CDI) (Nood et al. 2013). Moreover, given the importance of the gut microbiota for the efficacy of cancer therapy, FMT may be used to enhance the treatment of cancer patients. And indeed, the microbiota of cancer patients who have responded well to therapy can be used to transfer these therapeutic benefits to others (Baruch et al. 2021; Davar et al. 2021). Studies in humans are still rare and often have limited power, but they suggest that it may be possible to tailor patients to therapy rather than the other way around. A successful FMT results in a persistent shift in the recipient’s gut microbiome toward the composition of the donor’s microbiota (Davar et al. 2021). Therefore, understanding the ability of the graft to colonize is an important area of future research. We do not have much data on FMT in cancer yet, but studies on CDI suggest that different types of FMT have varying degrees of success (Pomares Bascunana et al. 2021), which opens up new avenues for exploration. Several specific bacteria are beginning to be associated with good or poor response to ICI (Table 1), but other members of the microbiota are much less understood. Interestingly, intestinal bacteriophages can regulate bacterial consortia and restoring the virome community may be just as important as the bacterial microbiome in treatment of CDI with FMT (Zuo et al. 2018). Similar principles may apply for FMT during ICI, making intestinal bacteriophages a promising focus of future research.

## Conclusion and future directions

Several microbes have long been associated with the development of cancer, with *H. pylori* being established as a known human carcinogen (IARC 2012). New bacterial species that can cause cancer are constantly being discovered, and there is currently ample evidence that the microbiota can influence all hallmarks of cancer, not only through local interactions but also through indirect mechanisms, reaching distant tissues. Recent advances in microbiology, immunology, and cancer biology have shed light on the crucial role of gut microbiota in tumor initiation, progression, and spread.

As we delve deeper into the intricate workings of host–microbe interactions, the potential for using this knowledge in clinical practice to improve cancer treatment and prevention becomes increasingly apparent. Several avenues of research are opening up for investigators in the basic and clinical sciences. In particular, research priority should be given to systematically investigating how the gut “immunological niche” interacts with nutritional and microbiological components and how the outcomes of these interactions reach distant tissues. Additionally, questions remain about how these early-life interactions between the host and the microbiota affect resistance to cancer later in life.

Although applications are still limited in the clinical arena, recent publications indicate that the gut microbiota can serve as potential biomarkers, therapeutic targets, or means to improve existing therapies by increasing their efficacy or decreasing their toxicity. Current research is now focused on identifying microbes predicting therapeutic outcome or identifying patient subgroups suitable for individualized therapies.

FMT presents a promising avenue for enhancing cancer therapy. However, before it can be used in clinical practice, we need to understand the underlying host-microbe interactions and tailor the approach to the specific cancer type and drug. While a single FMT is sufficient to correct acute dysbiosis in CDI, resolving long-term dysbiosis in chronic diseases such as cancer may require a different strategy. Therefore, in future studies, we may need to revise our approaches by focusing more on multiple FMTs or bacteriophage administration. The motivation behind this review was to combine concepts from different research areas to stimulate discussion among experts and open this complex and fascinating field to new researchers. We hope that this will ultimately accelerate progress toward new preventive and therapeutic interventions for cancer patients.

## Data Availability

Not applicable.

## Abbreviations

APC: Anaphase-promoting complex
ATBs: Antibiotics
Bcl-2: B cell lymphoma-2 protein
BEVs: Bacterial extracellular vesicles
CDI: *Clostridioides difficile* Infection
CRC: Colorectal cancer
CTL: Cytotoxic T cells
CTLA-4: Cytotoxic T-lymphocyte-associated protein 4
DCs: Dendritic cells
EMT: Epithelial-mesenchymal transition
FMT: Fecal microbiota transplantation
GF: Germ-free
HPV: Human papillomavirus
ICIs: Immune checkpoint inhibitors
IL: Interleukin
ILCs: Innate lymphoid cells
MAMP: Microorganism-associated molecular patterns
MDSCs: Myeloid-derived suppressor cells
MMPs: Matrix metalloproteinases
NF-κB: Nuclear factor kappa-light-chain-enhancer of activated B cells
NK: Natural killer
NSCLC: Non-small-cell lung cancer
PD-1: Programmed cell death protein 1
PD-L1: Programmed death ligand-1
PRR: Pattern recognition receptor
RNS: Reactive nitrogen species
ROS: Reactive oxygen species
RT: Radiotherapy
SCFAs: Short-chain fatty acids
STAT3: Signal transducer and activator of transcription 3
TAMs: Tumor-infiltrating macrophages
TGF: Transforming growth factor
Th: T helper
TLR: Toll-like receptor
TNF: Tumor necrosis factor
Tregs: Regulatory T cells
UC: Ulcerative colitis
